# Genetic polymorphisms associated with endothelial function in nonarteritic anterior ischemic optic neuropathy

**Published:** 2013-02-01

**Authors:** Tsutomu Sakai, Keigo Shikishima, Masato Matsushima, Hiroshi Tsuneoka

**Affiliations:** 1 Department of Ophthalmology, Jikei University School of Medicine, Tokyo, Japan; 2 Division of Clinical Research and development, Jikei University School of Medicine, Tokyo, Japan

## Abstract

**Purpose:**

To examine the relationship between nonarteritic anterior ischemic optic neuropathy (NAION) and genetic polymorphisms of enzymes influencing endothelial function.

**Methods:**

The subjects were 34 patients with NAION (mean age, 62.4 years old; 59% male) and 102 controls (mean age, 63.8 years old; 66% male). Genetic polymorphisms were investigated in three candidate genes associated with endothelial function: endothelin-1 (ET-1), angiotensin-converting enzyme (ACE), and methylenetetrahydrofolate reductase (MTHFR). The genotype distributions in the patients with NAION were compared with those in the controls.

**Results:**

There were no significant differences in the genotype distributions of the ACE I/D and MTHFR C677T polymorphisms between the NAION and control groups (p=0.261 and p=0.354, respectively), whereas the genotype distribution of the G/T (Lys198Asn) polymorphism of the ET-1 gene varied significantly between the groups (p=0.009). After adjusting for covariates, individuals with the TT genotype of the Lys198Asn polymorphism were more likely to develop NAION compared with those with the GG genotype (odds ratio=4.43, 95% confidence interval 1.33–14.73, p=0.015).

**Conclusions:**

We found an increased prevalence of a G/T polymorphism of the ET-1 gene in patients with NAION. Our data suggest that this polymorphism may be an important risk factor in developing NAION in the Japanese population.

## Introduction

Nonarteritic anterior ischemic optic neuropathy (NAION) is a disease caused by vascular insufficiency leading to optic nerve head ischemia, and characterized by acute, monocular, and painless visual loss [1]. Although risk factors such as diabetes mellitus [2-6], systemic hypertension [3-5,7], hyperlipidemia [7-9], and smoking [8,10,11] have been proposed, the pathogenesis of NAION is poorly understood. Hayreh et al. [12] have postulated an association with defective vascular autoregulation in the optic nerve head, while Potarazu [13] suggested that endothelial defects in the ocular circulation may impair the efficacy of endothelial-derived relaxation factors in NAION. Thus, impaired autoregulation and vasoconstriction of nutrient vessels supplying the optic nerve head may be involved in the pathogenesis of NAION.

Endothelin (ET)-1 is a 21 amino acid peptide that is a potent vasoconstrictor with an important role in the pathophysiology of the vascular system. Previous studies have shown that increased plasma ET-1 levels are associated with acute myocardial infarction [14], essential hypertension [15,16], and ischemic optic neuropathy [17], suggesting that an increased level of ET-1 leads to vascular dysregulation. Recent studies have also linked a G/T (Lys198Asn) polymorphism of the ET-1 gene with coronary artery disease [18] and variant angina, although there is no evidence that the ET-1 G/T polymorphism affects the gene product in a physiologic meaningful way [19].

Elevated serum homocysteine is another possible aggravating factor for vascular endothelial cell damage in NAION [20]. A C/T polymorphism at nucleotide position 677 (C677T) of the 5,10-methylenetetrahydrofolate reductase (MTHFR) gene has been reported [21], and the functional consequences of this polymorphism depend on the folate status in hemodialysis patients [22].

The renin-angiotensin system has important roles in atherosclerotic and cardiovascular disease, with effects ranging from endothelial dysfunction to vascular occlusion. Angiotensin-converting enzyme (ACE) governs conversion of angiotensin (Ang) I to Ang II and degradation of bradykinin. An insertion/deletion (I/D) polymorphism of the ACE gene (presence or absence of a 287 base pair insert in intron 16) significantly influences circulating and tissue ACE levels and results in greater production of Ang II [23]. This may be important in NAION, since the known actions of Ang II include vascular contraction. In contrast, a previous study [24] showed that the II genotype is a risk factor for NAION in younger male patients and that the I allele is likely a predisposing factor for NAION in younger men.

The relationship between NAION and genetic polymorphisms in enzymes influencing endothelial function is incompletely understood. In this study, candidate genes were selected based on published evidence of involvement in risk factors of NAION or suspected biochemical contribution to a disease pathway. We examined genetic polymorphisms of ET-1, ACE, and MTHFR in patients with NAION. These polymorphisms have been reported to be associated with ocular vascular diseases.

## Methods

Written informed consent was obtained from all subjects. The protocol was approved by the ethics committee at our institution, and the study was performed in accordance with the ethical standards laid down in the Declaration of Helsinki. The subjects were 34 patients with NAION (20 men and 14 women; mean age, 62 years old; age range, 48–91 years old). NAION was diagnosed by neuroophthalmologists following the criteria of the Ischemic Optic Neuropathy Decompression Trial Research Group [11]: 1) acute unilateral painless visual loss, 2) optic disc-related visual field defects, 3) presence of an afferent papillary defect, 4) optic disc edema, and 5) no other neurologic symptoms. The control group consisted of 102 consecutive patients (67 men and 35 women; mean age, 64 years old; age range 42–88 years old) who were referred to our institution for reasons other than NAION. All subjects were Japanese.

Subjects were defined as having diabetes mellitus if they were receiving treatment for insulin- or non-insulin-dependent diabetes mellitus. Hypertension was defined based on a history of hypertension with intake of antihypertensive drugs. Seven patients took these drugs at bedtime. Subjects were classified with hyperlipidemia if they were currently being treated for hyperlipidemia. The definition of smoking status included past and current smokers.

Venous blood was collected from all subjects. The whole blood samples (5 ml) from all the patients were collected in EDTA tube and stored at -20°C for fewer than one month storage. Genomic DNA was isolated from peripheral leukocytes according to a kit protocol (Qiagen, Hilden, Germany). Polymorphisms of the ET-1, ACE, and MTHFR genes were examined. Genotypes were determined with a fluorescence- or colorimetry-based allele-specific DNA primer assay system (Toyobo Gene Analysis, Tsuruga, Japan) [25]. Polymorphic regions of each gene were amplified with PCR with two allele-specific sense (or antisense) primers labeled at the 5′ end with fluorescein isothiocyanate or Texas red and an antisense (or sense) primer labeled at the 5′ end with biotin. Alternatively, polymorphic regions were amplified with two allele-specific sense (or antisense) primers and a biotin-labeled antisense (or sense) primer or with a sense primer and a biotin-labeled antisense primer. The sequences of the primers used in assays for the polymorphisms of ET-1, ACE, and MTHFR are shown in Table 1. Each reaction mixture included 20 ng of DNA, 5 pmol of each primer, 0.2 mmol/l of each deoxynucleotide triphosphate, 3 mmol/l of magnesium chloride, and 1 U of DNA polymerase buffer. The amplification protocol included an initial period of denaturation at 95 °C for 5 min, 35 to 45 cycles of denaturation at 95 °C for 30 s, annealing at 55 °C for 30 s, extension at 72 °C for 30 s, and a final period of extension at 72 °C for 2 min.

**Table 1:** Primers for Polymerase Chain Reaction Amplification

No	Gene	SNP	Rs No.	Primer name	marker	Sequence
1	Endothelin-1	G5665T	rs5370	F(Gtype)	FITC	CCAAGCTGAAAGGCAXGC
				F(Ttype)	TxR	CCCAAGCTGAAAGGCAXTC
				R	Biotin	TCACATAACGCTCTCTGGAGG
2	ACEI/D	I/D	rs1799752	F(Itype)	FITC	CTCGATCTCCTGACCTCGTGATCC
				F(Dtype)	TxR	CCTGCTGCCTATACAGTCACTTTTATG
				R	Biotin	GATGTGGCCATCACATTCGTCAGAT
3	MTHFR	C677T	rs1801133	F(Ttype)	FITC	GAGAAGGTGTCTGCGGGAXTC
				F(Ctype)	TxR	GAAGGTGTCTGCGGGAXCC
				R	Biotin	GAATGTGTCAGCCTCAAAGAAA

To confirm the accuracy of genotyping with this method, DNA samples were subjected to PCR, followed by restriction-fragment-length polymorphism analysis or direct DNA sequencing of the PCR products. In each instance, the genotype determined with the allele-specific DNA-primer-probe assay was identical to that determined using the confirmatory methods.

Statistical analysis was performed using STATA 8.0 (College Station, TX). Logistic regression analysis was used to evaluate the independent effect of ET-1 mutations on the occurrence of NAION by adjusting for covariates such as diabetes mellitus, hypertension, hyperlipidemia, and smoking status. The ET-1 mutations and other covariates were coded with indicator variables. The criterion for significance was p<0.05.

## Results

The distribution of the three polymorphisms of ET-1, ACE I/D, and MTHFR in the case and control groups was under Hardy–Weinberg equilibrium. The genotype and allele frequencies of the ET-1 gene polymorphisms are shown in Table 2 and Table 3. A significantly higher frequency of the ET-1 TT genotype was found in patients with NAION than in controls (29% versus 9%, p=0.009). Regarding gender, there was a significant difference in the frequency of the TT genotype in male cases compared to the male controls (32% versus 6%, p=0.007), but no significant difference was found when the female cases were compared to the female controls (24% versus 14%, p=0.578). The frequency of homozygosity for the G/T variant of the ET-1 gene was also significantly higher in patients with NAION than in the controls (44% versus 31%, p=0.04). Regarding gender, there was no significant difference in the frequency of homozygosity for the G/T variant of the ET-1 gene between the two groups for men and women (p=0.065 and p=0.262, respectively).

**Table 2:** The genotype frequencies of ET-1 gene polymorphisms in control and NAION.

Genotype	All subjects		Males		Females	
	Controls	NAION	Controls	NAION	Controls	NAION
G/T polymorphism	No (%)	No (%)	No (%)	No (%)	No (%)	No (%)
GG	47 (46)	14 (42)	31 (46)	8 (42)	16 (46)	6 (40)
GT	46 (45)	10 (29)	32 (48)	5 (26)	14 (40)	5 (36)
TT	9 (9)	10 (29)	4 (6)	6 (32)	5 (14)	4 (24)

**Table 3:** The allele frequencies of ET-1 gene polymorphisms in control and NAION.

Allele	All subjects		Males		Females	
	Controls	NAION	Controls	NAION	Controls	NAION
G/T polymorphism	No (%)	No (%)	No (%)	No (%)	No (%)	No (%)
G	140 (69)	38 (56)	94 (70)	21 (55)	46 (66)	17 (57)
T	64 (31)	30 (44)	40 (30)	17 (45)	24 (34)	13 (43)

Multivariable logistic regression analysis showed that the TT genotype of the ET-1 G/T polymorphism emerged as a significant risk factor for NAION after adjustments for age, diabetes mellitus, hypertension, hyperlipidemia, and tobacco smoking (Table 4). The odds ratio was 4.43 with the 95% confidence interval (CI) ranging from 1.33 to 14.73 (p=0.015).

**Table 4:** Logistic Regression Analysis.

Risk factors logistic regression model for each risk factor separately	Odds ratio	95% Confidence Interval	p value
Diabetes mellitus (yes/no)	2.99	1.09–8.22	0.033
Hypertension (yes/no)	0.78	0.30–2.00	0.605
Hyperlipidemia (yes/no)	2.33	0.95–5.72	0.064
Tobacco smoking (yes/no)	1.09	0.46–2.59	0.842
ET-1 TT (TT/GT)	4.43	1.33–14.73	0.015

The genotype and allele frequencies of the ACE and MTHFR gene polymorphisms are shown in Table 5, Table 6, Table 7, and Table 8. For genotype and allele distributions of ACE and MTHFR polymorphisms, there were no significant differences between the two groups of all subjects (ACE: p=0.261 and p=0.209, MTHFR: p=0.354 and p=0.221). Regarding gender, there was also no significant difference in the genotype and allele distributions of the ACE and MTHFR polymorphisms between the two groups for men and women (ACE genotype: p=0.665 and p=0.296, ACE allele: p=0.315 and p=0.262, MTHFR genotype: p=0.094 and p=0.894, MTHFR allele: p=0.084 and p=0.437). As a result, no association between these two polymorphisms with NAION occurrence was found.

**Table 5:** The genotype frequencies of ACE gene polymorphisms in control and NAION.

Genotype	All subjects		Males		Females	
	Controls	NAION	Controls	NAION	Controls	NAION
I/D polymorphism	No (%)	No (%)	No (%)	No (%)	No (%)	No (%)
II	42 (41)	15 (44)	27 (40)	9 (45)	15 (43)	6 (43)
ID	47 (46)	18 (53)	32 (48)	10 (50)	15 (43)	8 (57)
DD	13 (13)	1 (3)	8 (12)	1 (5)	5 (14)	0 (0)

**Table 6:** The allele frequencies of ACE gene polymorphisms in control and NAION.

Allele	All subjects		Males		Females	
	Controls	NAION	Controls	NAION	Controls	NAION
I/D polymorphism	No (%)	No (%)	No (%)	No (%)	No (%)	No (%)
I	131 (64)	48 (71)	86 (64)	28 (70)	46 (66)	17 (57)
D	73 (36)	20 (29)	48 (36)	12 (30)	24 (34)	13 (43)

**Table 7:** The genotype frequencies of MTHFR gene polymorphisms in control and NAION.

Genotype	All subjects		Males		Females	
	Controls	NAION	Controls	NAION	Controls	NAION
C677T polymorphism	No (%)	No (%)	No (%)	No (%)	No (%)	No (%)
CC	21 (21)	11 (32)	14 (21)	9 (45)	7 (20)	2 (14)
CT	56 (55)	15 (44)	37 (55)	7 (35)	19 (54)	8 (57)
TT	25 (24)	8 (24)	16 (24)	4 (20)	9 (26)	4 (29)

**Table 8:** The allele frequencies of MTHFR gene polymorphisms in control and NAION.

Allele	All subjects		Males		Females	
	Controls	NAION	Controls	NAION	Controls	NAION
C677T polymorphism	No (%)	No (%)	No (%)	No (%)	No (%)	No (%)
C	98 (48)	37 (54)	65 (49)	25 (63)	33 (47)	12 (43)
T	106 (52)	31 (46)	69 (51)	15 (37)	37 (53)	16 (57)

## Discussion

In this study, we found a significant association between the G/T polymorphism of the ET-1 gene and the occurrence of NAION in Japanese subjects. To the best of our knowledge, this is the first report of a positive association between an ET-1 gene polymorphism and NAION. In a previous study, we showed that patients with NAION have higher circulating levels of ET-1 than controls [17]. This result prompted us to investigate the effect of the ET-1 gene polymorphism on NAION. In the current study, the G/T polymorphism of ET-1 emerged as a significant risk factor for occurrence of NAION after adjustment for other factors in multivariate analysis. The pathogenic significance of the association of the ET-1 G/T polymorphism with NAION is unknown. However, some studies have suggested that the T allele is associated with increased plasma concentrations of ET-1 [26,27], and it has been proposed that vasospasms may be responsible for NAION [28,29]. Hayreh speculated that acute blood loss, a condition associated with NAION, could increase the release of endogenous vasoconstrictor agents, leading to vasoconstriction and NAION [1]. Collectively, these results suggest that the homozygous G/T variant of the ET-1 gene is a risk factor for developing NAION.

Analysis of a multifactorial disease requires that several coexisting and risk-modifying factors be taken into account. Hypertension, diabetes mellitus, and tobacco smoking have been related to NAION [1,10], and the G/T polymorphism of ET-1 has been associated with hypertension and diabetes mellitus [26,27,30-34]. In the current study, we examined histories of hypertension, diabetes mellitus, and smoking for inclusion with other factors (age, sex, and history of hyperlipidemia) as moderator variables; however, the association of the ET-1 G/T polymorphism with NAION did not change before and after adjustment for hypertension, diabetes mellitus, and smoking. Interestingly, only diabetes mellitus seemed to be a risk factor for development of NAION, as also found in previous studies [2,35].

In contrast to the ET-1 polymorphism, we found no positive association of development of NAION with the I/D polymorphism of the ACE gene or the C677T polymorphism of the MTHFR gene. Many reports have shown that the ACE I/D polymorphism is significantly associated with subclinical structural arteriolar changes [36], diabetic retinopathy [37,38], and hypertensive retinopathy [39], and that the MTHFR C677T polymorphism is significantly associated with retinal vein occlusion [40]. These findings suggest that ACE and MTHFR activity is mainly associated with retinal vascular diseases. Therefore, the ACE I/D and MTHFR C677T polymorphisms may affect a mechanism that promotes retinal vessel damage.

An important limitation of our study is the small number of cases. However, the distribution of the G/T genotype in the Japanese patients in our study is similar to the distribution found in previous studies [31,32] in a large number of Japanese subjects. Thus, we are confident that the frequencies of the GG, GT, and TT genotypes were accurately determined. However, examination of a larger number of cases is required to substantiate the connection between the ET-1 gene polymorphism and NAION. Another limitation is that we did not consider nocturnal hypotension, the most important risk factor for NAION. Thus, the association between the ET-1 gene polymorphism and nocturnal hypotension requires further examination. Within these limitations, we conclude that a G/T polymorphism in the ET-1 gene occurs with high prevalence in patients with NAION, and that this polymorphism may be an important risk factor for developing NAION in the Japanese population.
